# Prognostic Significance of Cystic Biliary Dilatation in Primary Sclerosing Cholangitis

**DOI:** 10.5152/tjg.2026.26055

**Published:** 2026-03-25

**Authors:** Yavuz Emre Parlar, Bengi Öztürk, Onur Keskin, Sabir İsrafilov, Taylan Kav, Cenk Sökmensüer, Ahmet Bülent Doğrul, Erkan Parlak

**Affiliations:** 1 Department of Gastroenterology, Sincan Training and Research Hospital, Ankara, Türkiye; 2 Department of Gastroenterology, Hacettepe University Faculty of Medicine, Ankara, Türkiye; 3 Department of Gastroenterology, TOBB University of Economics and Technology Hospital, Ankara, Türkiye; 4 Department of Medical Pathology, Hacettepe University Faculty of Medicine, Ankara, Türkiye; 5 Department of General Surgery, Hacettepe University Faculty of Medicine, Ankara, Türkiye

**Keywords:** Cholangitis, endoscopic retrograde cholangiopancreatography, percutaneous transhepatic biliary drainage, primary sclerosing cholangitis

## Abstract

**Background/Aims:**

Primary sclerosing cholangitis (PSC) is a chronic, progressive disease characterized by inflammatory and fibrotic strictures in the intrahepatic and/or extrahepatic bile ducts. Typical cholangiographic findings include multifocal, annular, short bile duct strictures with minimal upstream dilatation. However, in some patients, this dilatation progresses to cystic dimensions. The current study aimed to investigate whether the clinical features and prognosis of patients with cystic dilatation (CD) of the bile ducts differ from those of patients with classic cholangiographic PSC.

**Materials and Methods:**

Demographic data, laboratory and clinical findings, and prognostic outcomes of patients with CD of the bile ducts who underwent endoscopic retrograde cholangiopancreatography (ERCP) and/or percutaneous transhepatic biliary drainage at the hospital between 2017 and 2023 were compared with those of patients without CD.

**Results:**

Of the 37 patients, 5 (13.5%) had cystic biliary dilatation. Median follow-up was 24 months (range, 12-62) in the CD group and 20 months (range, 8-52) in the non-cystic group. Patients with CD had a higher frequency of cholangitis (median attacks per patient, 1.6 vs. 0.3; *P *< .001), a greater proportion of recurrent cholangitis (4/5 [80%] vs. 7/32 [21.8%]; *P*= .038), an increased risk of end-stage liver disease (3/5 [60%] vs. 5/32 [15.6%]; *P*= .025), and higher mortality (1/5 [20%] vs. 2/32 [6.25%]; *P*= .045). Median transplant-free survival was significantly shorter in the cystic group (7.1 years [95% CI, 1-9] vs. 19.5 years; *P*= .017).

**Conclusion:**

Cystic dilatation appears to be a clinically relevant prognostic feature in patients with primary sclerosing cholangitis, however, confirmation in larger studies is warranted.

**Main Points**

- Cystic biliary dilatation represents a distinct cholangiographic phenotype in primary sclerosing cholangitis that is associated with a more aggressive disease course.
- Patients with cystic biliary dilatation experience higher rates of recurrent cholangitis, repeated biliary interventions, and progression to end-stage liver disease.
- The presence of cystic biliary dilatation is associated with significantly reduced transplant-free survival and may serve as a clinically relevant prognostic marker in primary sclerosing cholangitis.

## Introduction

Primary sclerosing cholangitis (PSC) is characterized by progressive inflammatory and fibrotic strictures in the intrahepatic and/or extrahepatic bile ducts.^1^ Primary sclerosing cholangitis often presents with cholestasis, and disease progression may ultimately lead to biliary cirrhosis, liver failure, and cholangiocarcinoma. The classic cholangiographic signature of PSC is characterized by multifocal, annular, and short strictures with relatively limited upstream biliary dilatation.^2^ However, within the spectrum of this complex disorder, some patients exhibit bile duct dilatation that extends to cystic dimensions.

Primary sclerosing cholangitis is known for its unpredictable course, and its prognosis can vary widely among individuals. Factors influencing prognosis in PSC include the extent and severity of bile duct involvement, presence of complications such as cirrhosis, and development of associated conditions like inflammatory bowel disease (IBD).^3^

The available literature on cystic dilatation in PSC (PSC with CD) remains limited, with only small case series suggesting a potential association with unfavorable clinical outcomes, including increased risk of cholangiocarcinoma, need for liver transplantation, and higher mortality.^4^ The current study aimed to determine whether the clinical features and long-term prognostic outcomes of PSC patients with cystic bile duct dilatation differ from those of patients with classical cholangiographic presentation of PSC.

## Materials and Methods

### Study Design and Patient Selection

Retrospective data obtained from patients with PSC who underwent endoscopic retrograde cholangiopancreatography (ERCP) and/or percutaneous transhepatic biliary drainage (PTBD) at Hacettepe University Hospital, a tertiary referral center in Ankara, Türkiye between January 2017 and November 2023 were collected. Ethical committee approval was received from the Ethics Committee of University of Hacettepe (Approval no: SBA 24/049, Date: January 9, 2024). Written informed consent was waived by the local ethics committee due to the retrospective design of the study.

In the current study, PSC was defined according to the guidelines of the European Association for the Study of the Liver as chronic elevation of serum cholestatic liver enzymes together with typical cholangiographic features on magnetic resonance cholangiopancreatography (MRCP), or ERCP, and the absence of secondary causes of sclerosing cholangitis. Liver MRI with MRCP was available in all patients and used for baseline biliary assessment and follow-up evaluation. Endoscopic retrograde cholangiopancreatography was performed for therapeutic indications, including treatment of dominant bile duct strictures and management of biliary infection (recurrent cholangitis), after prior evaluation with liver MRI and MRCP.

There is no universally accepted size-based criterion for cystic biliary dilatation in PSC. Accordingly, CD was defined as a focal, nonuniform saccular or fusiform expansion of the bile ducts that was disproportionate to adjacent duct segments. Such dilatation was not attributable to simple upstream dilatation secondary to a stricture and was identified by localized outpouching with loss of ductal parallelism.^5^ This definition was based on cholangiographic results obtained from liver MRI with MRCP, and/or ERCP images used for complementary assessment during therapeutic biliary interventions by an experienced gastroenterologist.

Inflammatory bowel disease was diagnosed on the basis of colonoscopic findings and pathological examinations. The diagnosis of cholangiocarcinoma was confirmed through radiological imaging during the follow-up of patients with PSC, along with cytology and/or biopsy samples obtained from the biliary tract during ERCP when suspicion was raised. Liver cirrhosis was defined as the presence of portal hypertension, evidenced by splenomegaly, ascites, varices, or hepatic encephalopathy, along with compatible imaging findings and thrombocytopenia (< 100 000/µL).

Patients with choledochal cyst, Caroli disease, polycystic liver disease, IgG4-related PSC, and biliary obstruction (such as gallstones and cholangiocarcinoma) causing CD of the biliary ducts were excluded from the study.

### Data Collection and Laboratory Analysis

Demographic information (age and sex), clinical features (presenting symptoms and IBD status), laboratory parameters (alanine aminotransferase, aspartate aminotransferase, alkaline phosphatase, gamma-glutamyl transfarase, and total and direct bilirubin), and imaging findings were collected from the electronic hospital database. These baseline variables were compared between patients with PSC and cystic biliary dilatation and those without CD. Patients were grouped on the basis of the occurrence of cystic biliary dilatation during follow-up, as cystic biliary dilatation was not considered a baseline characteristic but a disease phenotype developing over time. The interval between PSC diagnosis and the first detection of CD was recorded individually to minimize potential lead-time or immortal-time bias.

### Endoscopic and Percutaneous Interventions

In patients with CD, during endoscopic procedures, following dilation of the stricture, 7-Fr and/or 10-Fr plastic stents were placed and left in place for 2 months before removal, according to operator preference. Only dilation or short-term stenting was performed in patients with a classically relevant stricture. In patients who underwent PTBD, following stricture dilation, the internal–external drain was maintained until adequate bile flow was achieved and subsequently removed.

### Outcome Measures and Follow-up

The primary outcome of this study was transplant-free survival, defined as the time from PSC diagnosis to death or liver transplantation.

Secondary outcomes included disease progression parameters such as symptomatic recurrent CD, number of cholangitis attacks, need for repeated therapeutic interventions, development of cirrhosis, development of cholangiocarcinoma, overall survival, and mortality rates. For all patients, follow-up and survival analyses were anchored to the date of PSC diagnosis.

This retrospective study was conducted in accordance with ethical principles, and approval for the review of radiological and clinical data was obtained from the institutional review board (Institutional Ethics Committee, Protocol No: SBA 24/049, Approval Date: January 9, 2024).

### Statistical Analysis

Descriptive statistics are presented as mean ± SD, median (range), or percentages. Continuous variables were compared using the Wilcoxon–Mann–Whitney *U-*test. For categorical variables, the chi-square test was used, and the Fisher’s exact test was applied when deemed appropriate. The median follow-up duration for the patients was determined from the date of PSC diagnosis to the last follow-up.

To estimate cumulative survival from the date of PSC diagnosis, Kaplan–Meier survival analysis was performed. Median survival times were calculated for patients with and without CD with respect to the outcomes of liver transplantation and death, and survival curves were compared using the log-rank test. Hazard ratios for death or liver transplantation were calculated using a multivariate Cox proportional hazards model adjusted for age at PSC diagnosis, presence of IBD, baseline liver biochemistry, and baseline cirrhosis. All statistical analyses were performed using IBM SPSS Statistics, version 23 (IBM SPSS Corp.; Armonk, NY, USA), and a *P* value <.05 was considered statistically significant.

## Results

**Figure 1.**
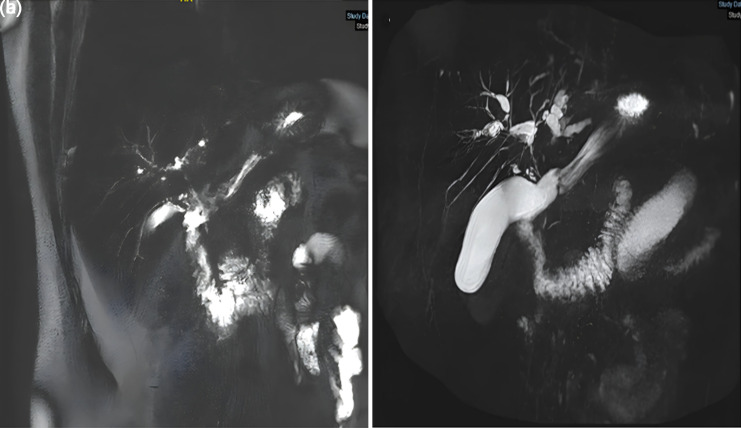
In a 70-year-old patient (patient no. 5), the initial magnetic resonance cholangiopancreatography showed no cystic biliary dilatation (a). However, magnetic resonance cholangiopancreatography performed 4 years later revealed cystic dilatation in the bile ducts (b). (This image was enhanced using AI-based upscaling for visualization purposes only; no new structures were introduced.)

The clinical characteristics at the time of diagnosis in patients with PSC with and without CD are presented in Table 1. Patients with CD are highlighted in Table 2. Five of the 37 patients (13.5%) had cystic biliary dilatation. All 5 patients with CD were male, and the time between PSC diagnosis and detection of CD (in months) was 48, 60, 36, 10, and 48. Figure 1 shows the absence of cystic biliary dilatation on the initial MRCP, with CD appearing after 4 years (patient no 5). In the group without CD, 65.7% of the patients (n = 21) were male, and the median follow-up time from PSC diagnosis was 20 months (range: 8-52 months). In patients with PSC and CD , 60% had concomitant IBD (all ulcerative colitis [UC]), compared to 53.12% in the non-cystic group (40.6% with UC and 12.5% with Crohn's disease [CD]). In both study and control groups, all patients received ursodeoxycholic acid at a dose of 20 mg/kg.

**Figure 2.**
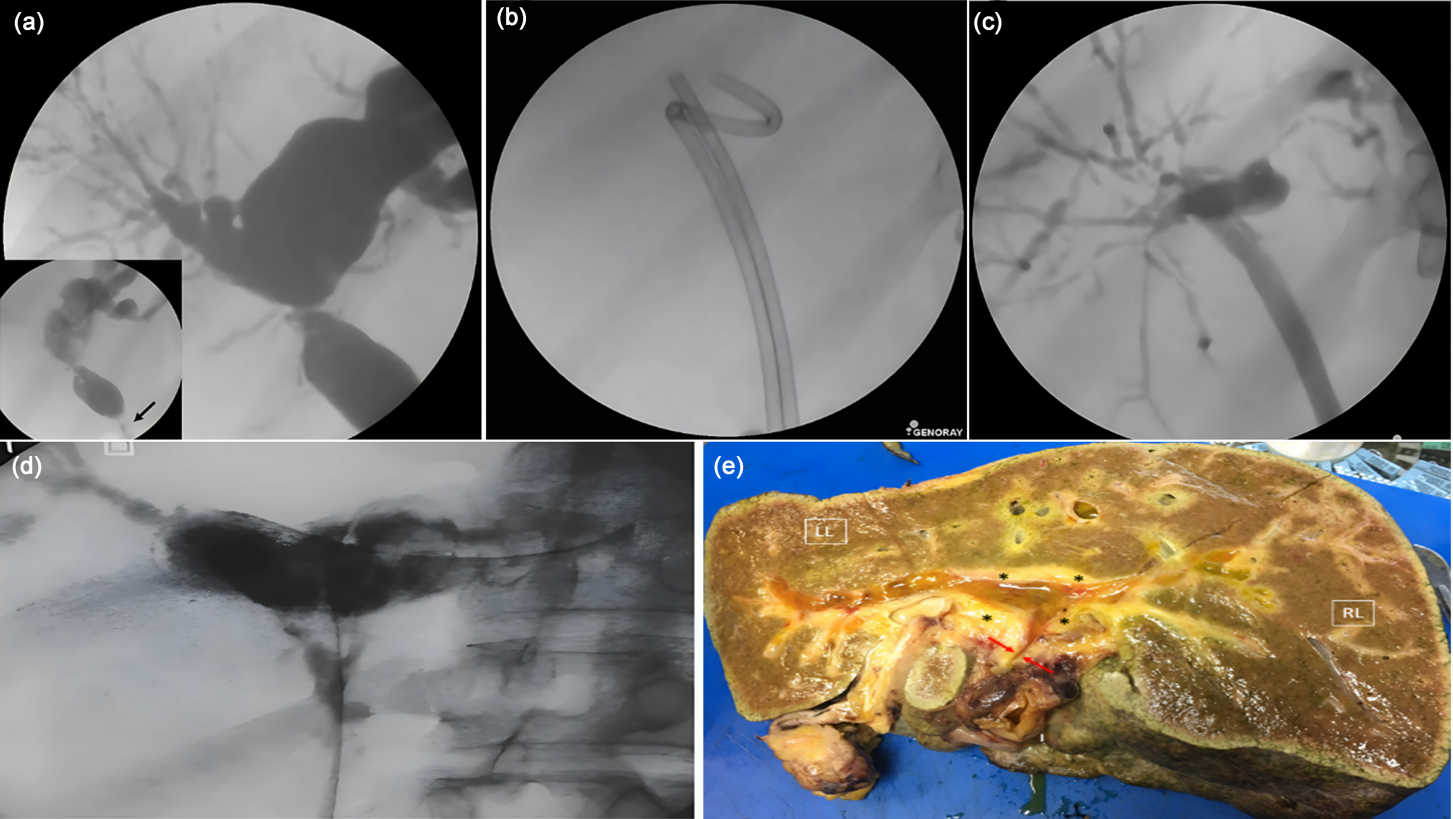
(a) Endoscopic retrograde cholangiopancreatography images of a 32-year-old primary sclerosing cholangitis patient (patient no: 2). (b) Initial cholangiography showed strictures at the hilum and distal common bile duct (arrows), along with prestenotic cystic dilatations in common bile duct and hilum. (c) Two 10 Fr stents were placed across the stricture. Follow-up cholangiography at 2 months (d) showed resolution of the cystic dilatations. Approximately 26 months later, recurrent symptomatic cystic dilatation (e) developed in the patient. Explant liver examination revealed fibrotic wall thickening in the cystic areas (stars) and strictures at the hilum (arrows).

Endoscopic retrograde cholangiopancreatography was performed in 4 of the 5 patients with PSC and CD (patients 1, 2, 3, and 4). Percutaneous transhepatic biliary drainage was performed in 2 patients (patients 4 and 5) due to endoscopic insufficiency and altered surgical anatomy. Cystic dilatation regressed in all patients who underwent endoscopic or percutaneous decompression (shown in Figure 2a-c).

Follow-up data are presented in Table 3. During follow-up, symptomatic recurrent CD developed in 3 patients (after 3 months, 5 years, and 26 months). Repeat ERCP and/or PTBD procedures were performed in all patients. One patient was being followed up with percutaneous drainage, and no recurrence developed in the other patient after 62 months of follow-up (patient 4). During follow-up after the index biliary intervention, the average number of cholangitis attacks per patient was significantly higher in the PSC with CD group (*P* < .001). In addition, a significantly greater proportion of patients with CD required repeat ERCP and/or PTBD during follow-up compared to those without CD (80% vs. 50%, *P* = .038).

When comparing progression rates to end-stage liver disease, a significantly higher rate was observed in the PSC with CD group compared to the PSC without CD group (60% vs. 15.6%, *P*: .028). During the follow-up period, only 1 patient (3.12%) in the PSC without CD group was diagnosed with cholangiocarcinoma. No statistically significant difference was observed between the groups in terms of the risk of cholangiocarcinoma development (*P*= .865).

**Figure 3.**
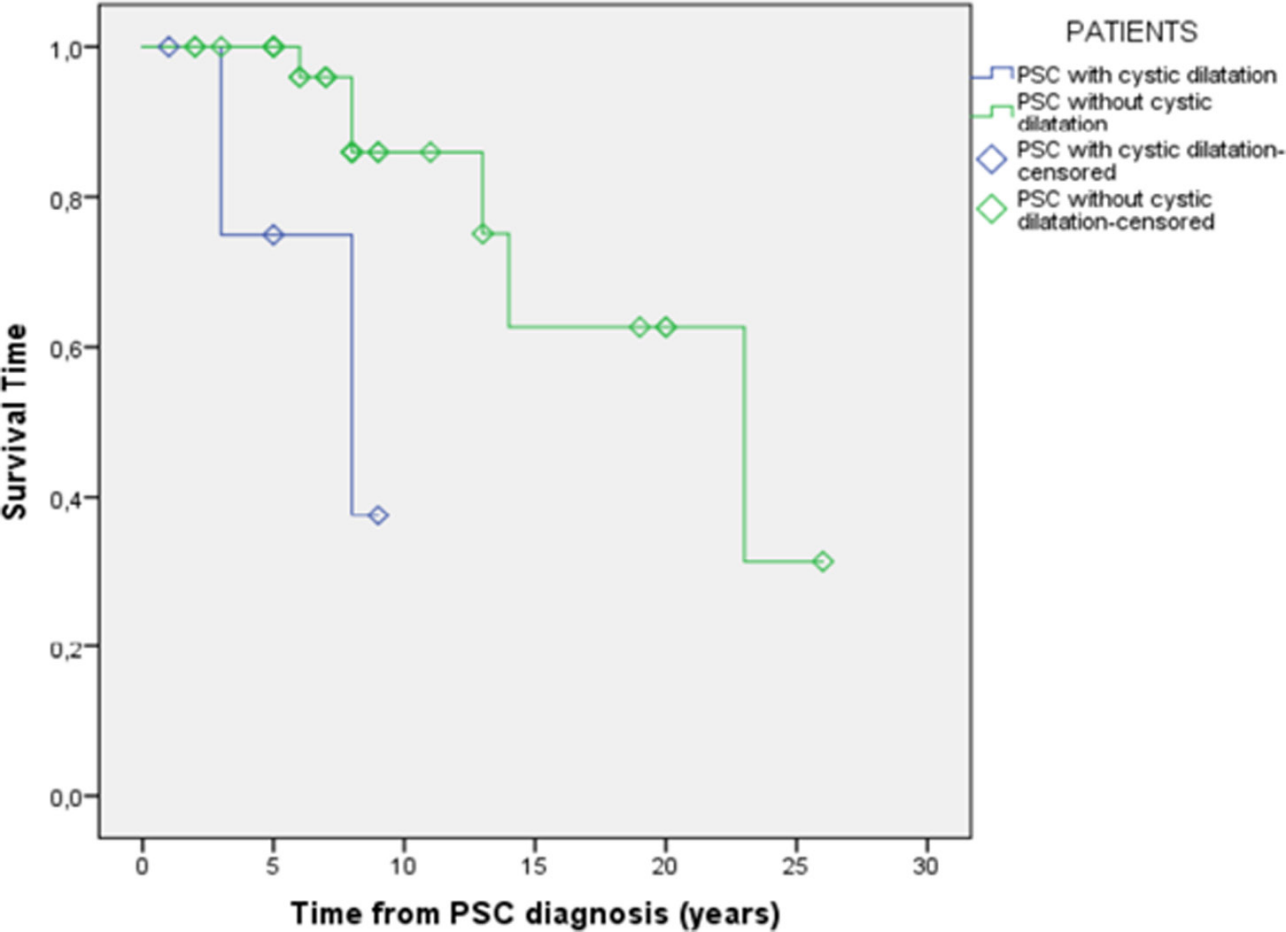
Kaplan-Meier survival analysis of primary sclerosing cholangitis patients with and without cystic dilatation.

In patients with PSC and CD, the median survival time from diagnosis to death or liver transplantation was 7.1 years (95% CI, 1-9 years). When compared to 32 PSC without CD patients (7.1 vs. 19.5 years, *P*= .017), the survival time was significantly shorter in the group of patients with CD (shown in Figure 3). On multivariate Cox regression, CD was associated with a 3.8-fold increased hazard of death or transplant (HR 3.8, 95% CI 1.1-13.2, *P*= .035).

## Discussion

Primary sclerosing cholangitis is a chronic cholestatic liver disease characterized by inflammation, fibrosis, and narrowing of bile ducts. In the current study, we investigated the clinical and prognostic significance of cystic biliary dilatation in patients with PSC and found that its presence was associated with a more severe disease course, including recurrent cholangitis, increased need for biliary interventions, and reduced transplant-free survival.

Due to the sclerotic nature of PSC, marked dilatation is generally absent in intrahepatic ducts.^6^ The presence of biliary PSC with CD has been investigated for its potential prognostic effects. In the current study, biliary CD was identified in 5 out of 37 patients (13.5%), which is higher than the prevalence reported in previous studies, including a European cohort by El Mouhadi et al. (7%) and the study by Nguyen et al. (7.3%).^5^ The higher rate of CD observed in the current study may be attributed to the tertiary care status of the hospital and the fact that the study cohort consisted solely of patients who underwent endoscopic treatment.

The exact mechanism underlying recurrent PSC with CD remains an area of ongoing investigation; however, the current study suggests multiple potential contributing factors. Severe biliary wall inflammation, ulceration, and epithelial necrosis may weaken the bile duct walls, making them more prone to dilation.^5^ Recurrent infections, which are commonly observed in PSC, can exacerbate tissue injury and promote cyst formation.^7^ Resolution of CD was observed in all patients on the 2-month-follow-up imaging after stenting, but recurrence of cystic enlargement was observed in 3 patients during subsequent follow-up. This result suggests that cystic enlargement may be prestenotic (Figure 2a, e). In another previously reported patient who was not included in this series, it was also observed that CD regressed after stenting, further supporting the idea that cystic enlargement is prestenotic.^8^ The recurrence of dilatation at the same points may imply that periductal fibrosis, a part of the pathogenesis of PSC, is not uniformly distributed at every point, and that cystic enlargement may occur in areas where fibrosis is relatively less pronounced.

There are 8 studies in the literature reporting PSC with CD.^4^^,^^5^^,^^8^^-^^13^ However, only a subset of these studies has specifically addressed the prognostic significance of CD. The current study revealed a significant association between biliary CD and recurrent cholangitis. The number of patients in the PSC with CD group who experienced recurrent acute cholangitis attacks and required repeat ERCP procedures is significantly higher compared to that in the PSC without CD group. This aligns with the results of the previous studies. Harrison et al. examined liver biopsy results from patients with PSC who underwent liver transplantation and reported that patients with CD, especially those exceeding 1 cm, frequently experienced recurrent cholangitis.^14^ Similarly, Moctezuma-Velázquez et al. described a patient with CD in the right intrahepatic bile ducts who continued to have recurrent cholangitis despite stenting and antibiotics, ultimately requiring surgical resection.^10^ The increased risk of cholangitis in patients with cystic PSC may be associated with elevated bile stasis, bacterial overgrowth, and chronic inflammation within the cystic structures.

The current study demonstrated a strong correlation between biliary CD and progression to end-stage liver disease in patients with PSC. Cystic outpouchings contribute to bile stasis, promoting the prolonged exposure of the biliary epithelium to toxic bile acids and inflammatory mediators. This environment facilitates ongoing tissue damage, triggering fibro-inflammatory processes that culminate in biliary cirrhosis.^11^ In 1 study, nearly half of the patients with PSC and CD presented with cirrhosis at the time of diagnosis.^5^

Furthermore, the current study does not identify a significant difference in the risk of cholangiocarcinoma development between PSC with CD and PSC without CD. Although only one patient in the PSC without CD group is diagnosed with cholangiocarcinoma during the follow-up period, caution is warranted in drawing definitive conclusions because of the small sample size and short follow-up duration. Additionally, patients either died or underwent liver transplantation, further limiting long-term observations. Studies focusing on patients with PSC and CD do not explicitly indicate that CD increases the risk of developing cholangiocarcinoma.^15^ However, in a study conducted by Ludwig et al., the pathological examination of a PSC with CD patient revealed epithelial hyperplasia, suggesting that it could be a precursor lesion for neoplastic transformation.^11^

Treatment outcomes in patients with PSC and CD remain suboptimal. While endoscopic therapies, such as balloon dilation and stent placement, can temporarily relieve symptoms, CD often recurs.^10^ In this cohort, all patients with CD who experienced cholangitis underwent repeat ERCP and/or PTBD during the follow-up. The high rate of interventions mirrors previous reports, highlighting the limitations of current therapeutic strategies. Liver transplantation remains the definitive treatment for end-stage disease; however, PSC recurrence in the allograft is a concern, necessitating long-term follow-up.^16^

Additionally, the survival rate is significantly higher in the PSC without CD group compared to the PSC with CD group. In a single-center cohort, patients with PSC and CD accounted for 7.3% of the cohort and had a significantly worse prognosis, with a median survival of 10.7 years compared to 23.4 years in those without CD.^5^ The statistically significant difference in survival rate between the PSC with CD and PSC without CD groups underscores the potential prognostic value of biliary CD in predicting adverse outcomes. Unlike the larger cohort reported by Nguyen et al., the current study provided unique longitudinal evidence showing both regression and recurrence of CD following biliary decompression. Reappearance of cystic enlargement at the same anatomic sites suggested a reproducible pattern of segmental susceptibility, offering an additional clinical perspective that has not been highlighted in previous studies.

Distinguishing PSC with CD from other hepatobiliary diseases that may present with overlapping clinical and radiological features can be challenging. These conditions include Caroli's disease, choledochal cysts, and cholangiocarcinoma. Accompanying biliary strictures and liver parenchymal abnormalities, characteristic of other biliary symptoms of PSC, may aid in differentiating PSC with CD from Caroli's disease.^17^ The fluctuating course and later onset observed in PSC with CD may help to distinguish it from choledochal cysts. Furthermore, the absence of progression and lack of mass lesions on imaging can differentiate PSC with CD from cholangiocarcinoma.^18^ A comprehensive evaluation combining clinical, radiological, and histopathological findings is essential for an accurate diagnosis.

This study is limited by its retrospective, single-center design, and the relatively small number of PSC with CD cases, which may constrain generalizability. In addition, the exclusive inclusion of patients managed at a tertiary referral center and requiring ERCP or PTBD may have introduced selection bias toward more advanced disease. Follow-up duration is also limited, and currently there is no standardized definition of CD in the literature, potentially affecting reproducibility. Nevertheless, the observed associations are robust and statistically significant, suggesting that CD may represent a meaningful prognostic marker and warranting further investigation in large, prospective multicenter studies.

In conclusion, the findings suggest that biliary CD may represent a pre-stenotic cholangiographic pattern in PSC and may be associated with disease progression. Although the underlying mechanisms linking CD to adverse outcomes remain unclear, its presence can be considered during clinical evaluation and risk stratification. Further studies in larger cohorts are required to validate these observations and better define their prognostic relevance.

## Figures and Tables

**Table 1.** Demographic and Clinical Characteristics of PSC Patients with and without Cystic Dilatation

	**PSC with Cystic Dilatation**	**PSC Without Cystic Dilatation**	***P***
Number of patients, n (%)	5 (13.5)	32 (86.5)	
Gender, n (%)			
Female	0	11 (34.3)	.295
Male	5 (100)	21 (65.7)	**.01**
Age (median)	43	45	.205
PSC diagnosis age (median)	37	41	.109
Symptoms, n (%)			
Abdominal pain	5 (100)	14 (43.7)	**.046**
Jaundice	4 (80)	16 (50)	.348
Itching	5 (100)	10 (31.2)	**.007**
Weakness	5 (100)	15 (46.8)	.051
Concomitant IBD, n (%)			.774
UC	3 (60)	13 (40.6)	
CD	0	4 (12.5)	
Liver function tests			
ALT	56.6 ± 34.44	109.26 ± 92.96	.229
AST	57.20 ± 31.01	79.21 ± 50.06	.853
ALP	400 ± 302	364 ± 276	.758
GGT	238 ± 172	315 ± 389	.408
Total bilirubin	4.74 ± 5.98	4.58 ± 6.16	.964
Direct bilirubin	2.72 ± 3.65	2.53 ± 4.08	.902
Follow-up time (median, months)	24 (12-62)	20 (8-52)	

ALP, alkaline phosphatase; ALT, alanine aminotransferase; AST, aspartate aminotransferase; CD, Crohn’s disease; GGT, gamma-glutamyl transferase; IBD, inflammatory bowel disease; PSC, primary sclerosing cholangitis; UC, ulcerative colitis.

**Table 2.** Overview of PSC Patients with Cystic Dilatation: Demographics, Clinical Course, and Outcomes

	**Patient 1**	**Patient 2**	**Patient 3**	**Patient 4**	**Patient 5**
Gender	Male	Male	Male	Male	Male
Age, years	35	32	29	49	70
Time between PSC diagnosis and cystic dilatation (months)	48	60	36	10	48
Follow-up period (months)	60	48	12	62	24
UC (yes, no)	No	No	Yes	Yes	Yes
Outcome					
Cystic resolution	✓	✓	✓	✓	✓
Symptomatic recurrent cystic dilatation, months	60	26	*	None	3
Cirrhosis	✓	✓			✓
Liver transplantation		✓			
Exitus					✓

ERCP, endoscopic retrograde cholangiopancreatography; PSC, primary sclerosing cholangitis; PTBD, percutaneous transhepatic biliary drainage; UC, ulcerative colitis.

* Still being followed up with percutaneous drainage.

**Table 3.** Follow-up Data of PSC Patients with and without Cystic Dilatation

	**PSC with Cystic dilatation**	**PSC Without Cystic Dilatation**	***P***
Total number of cholangitis during the follow-up period (n)/number of cholangitis attacks per patient (median)	8/1.6	10/0.3	**<.001**
Number of patients with recurrent cholangitis, n (%)	4 (80)	7 (21.8)	**.029**
Total ERCP/PTBD (n)	13	82	.702
Liver cirrhosis, n (%)	3 (60)	5 (15.6)	**.025**
Cholangiocarcinoma, n (%)	0	1 (3.12)	.865
Liver transplant, n (%)	1 (20)	3 (9.3)	.087
Posttransplant follow-up period (months)	24	12	
Exitus, n (%)	1 (20)	2 (6.25)	**.045**

ERCP, endoscopic retrograde cholangiopancreatography; PSC, primary sclerosing cholangitis; PTBD, percutaneous transhepatic biliary drainage.

## Data Availability

The data that support the findings of this study are available on request from the corresponding author.

## References

[1] Rabiee A Silveira MG. Primary sclerosing cholangitis. Transl Gastroenterol Hepatol. 2021;6:29. (doi: 10.21037/tgh-20-266) PMC7829069 33824933

[2] Trivella J John BV Levy C. Primary biliary cholangitis: epidemiology, prognosis, and treatment. Hepatol Commun. 2023;7(6):e0179. (doi: 10.1097/HC9.0000000000000179) 37267215 PMC10241503

[3] Bowlus CL Arrivé L Bergquist A AASLD practice guidance on primary sclerosing cholangitis and cholangiocarcinoma. Hepatology. 2023;77(2):659 702. (doi: 10.1002/hep.32771) 36083140

[4] Genève J Dubuc N Mathieu D Cystic dilatation of intrahepatic bile ducts in primary sclerosing cholangitis. J Hepatol. 1990;11(2):196 199. (doi: 10.1016/0168-8278(90)90113-6) 2254629

[5] Nguyen L Cazzagon N Corpechot C Intrahepatic cystic biliary dilatation constitutes a significant prognostic factor in patients with primary sclerosing cholangitis. Eur Radiol. 2019;29(3):1460 1468. (doi: 10.1007/s00330-018-5697-3) 30159619

[6] Sirpal S Chandok N. Primary sclerosing cholangitis: diagnostic and management challenges. Clin Exp Gastroenterol. 2017;10:265 273. (doi: 10.2147/CEG.S105872) 29138587 PMC5680897

[7] European Association for the Study of the Liver. EASL Clinical Practice Guidelines on sclerosing cholangitis. J Hepatol. 2022;77(3):761 806. (doi: 10.1016/j.jhep.2022.05.011) 35738507

[8] Parlak E Köksal AŞ Dışıbeyaz S Unusual cholangiographic findings in a patient with primary sclerosing cholangitis: cystic dilatation. Turk J Gastroenterol. 2012;23(6):792 794. (doi: 10.4318/tjg.2012.0496) 23864458

[9] Kumar S. Peribiliary cysts mimicking primary sclerosing cholangitis and cholangiocarcinoma. Cureus. 2022;14(1):e21435. (doi: 10.7759/cureus.21435) PMC8860721 35223223

[10] Moctezuma-Velázquez C Saúl-Pérez A López-Méndez E. Quiste de la vía biliar y colangitis de repetición como manifestaciones iniciales de colangitis esclerosante primaria [Primary sclerosing cholangitis presenting as recurrent cholangitis and right hepatic duct outpouching]. Gac Med Mex. 2012;148(5):476 479. 23128889

[11] Ludwig J MacCarty RL LaRusso NF Intrahepatic cholangiectases and large-duct obliteration in primary sclerosing cholangitis. Hepatology. 1986;6(4):560 568. (doi: 10.1002/hep.1840060403) 3525365

[12] Theilmann L Stiehl A. Detection of large intrahepatic cholangiectases in patients with primary sclerosing cholangitis by endoscopic retrograde cholangiography. Endoscopy. 1990;22(1):49 50. (doi: 10.1055/s-2007-1010726) 2307131

[13] Goldwire FW Norris WE Koff JM An unusual presentation of primary sclerosing cholangitis. World J Gastroenterol. 2008;14(43):6748 6749. (doi: 10.3748/wjg.14.6748) 19034983 PMC2773322

[14] Harrison RF Hubscher SG. The spectrum of bile duct lesions in end-stage primary sclerosing cholangitis. Histopathology. 1991;19(4):321 327. (doi: 10.1111/j.1365-2559.1991.tb00046.x) 1937411

[15] Tabibian JH Ali AH Lindor KD. Primary sclerosing cholangitis, Part 2: Cancer risk, prevention, and surveillance. Gastroenterol Hepatol (N Y). 2018;14(7):427 432. 30166959 PMC6111497

[16] Shah YR Nombera-Aznaran N Guevara-Lazo D Liver transplant in primary sclerosing cholangitis: current trends and future directions. World J Hepatol. 2023;15(8):939 953. (doi: 10.4254/wjh.v15.i8.939) 37701917 PMC10494561

[17] Yonem O Bayraktar Y. Clinical characteristics of Caroli's disease. World J Gastroenterol. 2007;13(13):1930 1933. (doi: 10.3748/wjg.v13.i13.1930) 17461492 PMC4146968

[18] Patel T. Cholangiocarcinoma--controversies and challenges. Nat Rev Gastroenterol Hepatol. 2011;8(4):189 200. (doi: 10.1038/nrgastro.2011.20) 21460876 PMC3888819

